# Novel Laboratory Approaches in Heavy Chain Disease With Discordant Immunoglobulin Quantitation: A Case Report and Literature Review

**DOI:** 10.1002/jcla.70202

**Published:** 2026-03-19

**Authors:** Eleonora Longhi, Artan Çeka, Fabiola Olivieri, Marco Moretti, Jacopo Sabbatinelli

**Affiliations:** ^1^ Postgraduate School in Clinical Pathology and Clinical Biochemistry Università Politecnica Delle Marche Ancona Italy; ^2^ SOD of Laboratory Medicine Azienda Ospedaliero Universitaria Delle Marche Ancona Italy; ^3^ Department of Clinical and Molecular Sciences (DISCLIMO) Università Politecnica Delle Marche Ancona Italy; ^4^ Advanced Technology Center for Aging Research IRCCS INRCA Ancona Italy; ^5^ Clinic of Laboratory and Precision Medicine IRCCS INRCA Ancona Italy

**Keywords:** heavy chain disease, hematological malignancies, immunoglobulins, monoclonal gammopathies, protein analysis

## Abstract

**Background:**

Heavy chain disease (HCD) is a rare plasma‐cell neoplasm frequently linked to lymphoproliferative and autoimmune disorders. Clinical presentation varies widely, with no conventional signs or symptoms. The prognosis is frequently uncertain, and there is currently no typical diagnosis or treatment. Truncated immunoglobulins' heavy chains (HCs) identification and bound light chains (LCs) exclusion are diagnostic requirements. The initial approach is laboratory‐based, mostly from protein analysis, as serum protein electrophoresis (SPE), immunotyping (IT), and immunofixation (IFE) are usually the first diagnostic steps.

**Methods:**

A panel of tests resulted in low serum protein, so SPE, IT, and IFE were conducted. The findings established a spike's presence and γ‐HC isotype, but differential diagnosis from gammopathies with LCs' low reactivity to reagents required further testing. Consequently, a molecular weight separation gel and immunoselection were executed.

**Results:**

SPE showed a 29.4% (12.9 g/L) spike central to the γ‐fraction, while IT resulted in a subtraction in the IgG window, later verified by serum IFE. Immunoselection showed no LC‐HC link, excluding a gammopathy with difficult LC identification. The molecular weight gel revealed a 160 kDa band. This rare case of HCD presents unique complexity, also due to discordant IgG3–IgG quantitation, associated angioimmunoblastic lymphoma morphology, and EBV genome.

**Conclusion:**

The aim is to draw attention to the difficulties in diagnosing HCD and stress the importance of both advanced methods and a proper laboratory approach. This is necessary to ensure prompt and effective care for a rare condition, particularly with complex presentations like this case.

## Graphical abstract

In this study, we investigated a rare case of γ‐heavy chain disease (γ‐HCD) through an integrated laboratory approach. Routine protein analyses (serum protein electrophoresis, immunotyping, and serum/urine immunofixation) identified an IgG monoclonal component without detectable light chains. Subsequent immunoselection confirmed the absence of heavy chains–light chains association and suggested truncation in γ‐heavy‐chain fragments, supporting the diagnosis.

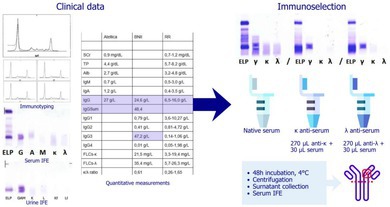

## 1 Introduction

Heavy chain disease (HCD) is a group of conditions with increased immunoglobulin heavy chains (HCs) production. These molecules, either in monomeric or dimeric conformation, have no association with a light chain (LC) counterpart, thus compromising the physiological function as effectors of humoral immunity [1].

Five types of mammalian heavy chains determine immunoglobulin classes; the most frequently involved in HCD are α, γ, and μ isotypes. The second defines Franklin's disease or gamma‐heavy chain disease (γ‐HCD), the first discovered in 1964 [2]. This condition is often associated with autoimmune diseases and lymphoproliferative disorders.

γ‐HCD is considered part of plasma cell neoplasms by the *WHO 5th Classification of Lymphoid Neoplasms* [3]; yet, not all cases may fall into a clear entity. Such conditions exhibit a broad spectrum in clinical, laboratory, and morphological presentation. The variable expression does not always allow for a clear classification and laboratory identification, furthering a delay in diagnosis and treatment [4].

Systemic symptoms such as anorexia, fatigue, fever, weight loss, associated with recurrent infections, lymphadenopathy, and organomegaly are exhibited by most patients. Autoimmune manifestations are also frequent [5].

Diagnosis is mainly laboratory‐based and requires identifying HCs without bound LCs in either serum or urine samples [4]. In this regard, serum protein electrophoresis (SPE), immunotyping (IT), and immunofixation (IFE) are the first steps to disease recognition.

Due to the wide range of manifestations, there is no set treatment protocol for γ‐HCD; instead, the specific presentation and lymphoproliferative disease determine the best course of action in a precision‐medicine approach [1].

We report a first‐time case of γ‐HCD and angioimmunoblastic lymphoma (AITL) with paradoxical IgG3 higher than total IgG. The latter is probably linked to an analytical phenomenon also investigated by Feugray et al. [6]. This issue may be related to the reagents' ability to quantify in either excess or defect due to changes in the target epitopes. Such changes are seen in altered immunoglobulin, such as the truncated heavy chain typical of HCD. It is up for further investigations whether HCs' conformation is bound to the lymphomagenic activity of EBV, known to frequently alter the expression of immunoglobulins' heavy chains.

## 2 Case Description

A 76‐year‐old male came to the attention of a medical facility for worsening and month‐recurring fatigue with global deterioration of health conditions. No remarkable medical history was recorded. Physical examination revealed splenomegaly and generalized edema. A basic panel of laboratory testing could not give a conclusive response, with all results falling within or close to the reference ranges. Imaging revealed numerous supradiaphragmatic plus subdiaphragmatic lymphadenopathies, including mediastinal and inguinal nodal involvement.

Suspecting a lymphoproliferative condition, surgical excision of an inguinal lymph node was performed. After recovery, the patient was discharged pending the pathology report. No further diagnostic procedures or disease‐related treatments were documented during the following weeks.

A month later, a local infection developed at the site of excision, so the patient was hospitalized with concern for impending sepsis at presentation. Upon admission, a broad panel of exams was performed. Overall, mild lympho‐monocytosis (Table 1), thrombocytopenia, increased inflammatory markers (PCR 5.04 mg/dL, procalcitonin 0.23 ng/mL), hematological indexes (β2‐microglobulin 19.8 μg/mL, LDH 406 U/L), and high EBV genome levels (39.099 UI/mL) with EBV IgG antibodies (Anti‐VCA, Anti‐EBNA) were detected. Deep wound cultures obtained from the surgical site were positive for *Staphylococcus aureus* and *Pseudomonas aeruginosa* . Blood cultures remained negative. The final pathology report confirmed the suspicion of an underlying angioimmunoblastic T‐cell lymphoma (AITL). Other laboratory findings are listed below, as the main investigations for this case are related to protein analysis.

**TABLE 1:** Full blood count and WBC differential.

	Day 1	RR
WBC	10.06 × 10^3^/mmc	4–10 × 10^3^/mmc
RBC	4.84 × 10^6^/mmc	4.5–5.5 × 10^6^/mmc
HGB	12.8 g/dL	12.5–17 g/dL
HCT	40%	40%–50%
MCV	83 fL	80–98 fL
MCH	26.4 pg	27–31 pg
MCHC	32 g/dL	32–36 g/dL
RDW CV	24.6%	11.5%–14.5%
PLT	133 × 10^3^/mmc	150–400 × 10^3^/mmc
Neutrophils	4.24 × 10^3^/mmc (42.2%)	1.9–7.7 × 10^3^/mmc
Lymphocytes	4.53 × 10^3^/mmc (45%)	1–4 × 10^3^/mmc
Monocytes	1.18 × 10^3^/mmc (11.7%)	0.1–1 × 10^3^/mmc
Eosinophils	0.01 × 10^3^/mmc (0.1%)	0.01–0.5 × 10^3^/mmc
Basophils	0.10 × 10^3^/mmc (1%)	< 0.1 × 10^3^/mmc

*Note:* Full blood count and differential leukocyte count parameters at presentation.

Abbreviation: RDW CV, red cell distribution width coefficient of variation.

### 2.1 Protein Analysis

After evidence of low serum protein (44 g/L) and albumin (27 g/L) with mild proteinuria (0.9 g/24 h), serum protein electrophoresis (SPE) was requested.

The patient's serum was first analyzed by capillary zone electrophoresis (CZE) performed by Sebia Capillarys 3 OCTA (Sebia S.A., Lisses, France), which revealed a broad spike central to γ‐fraction, involving 29.4% of total proteins.

**FIGURE 1:**
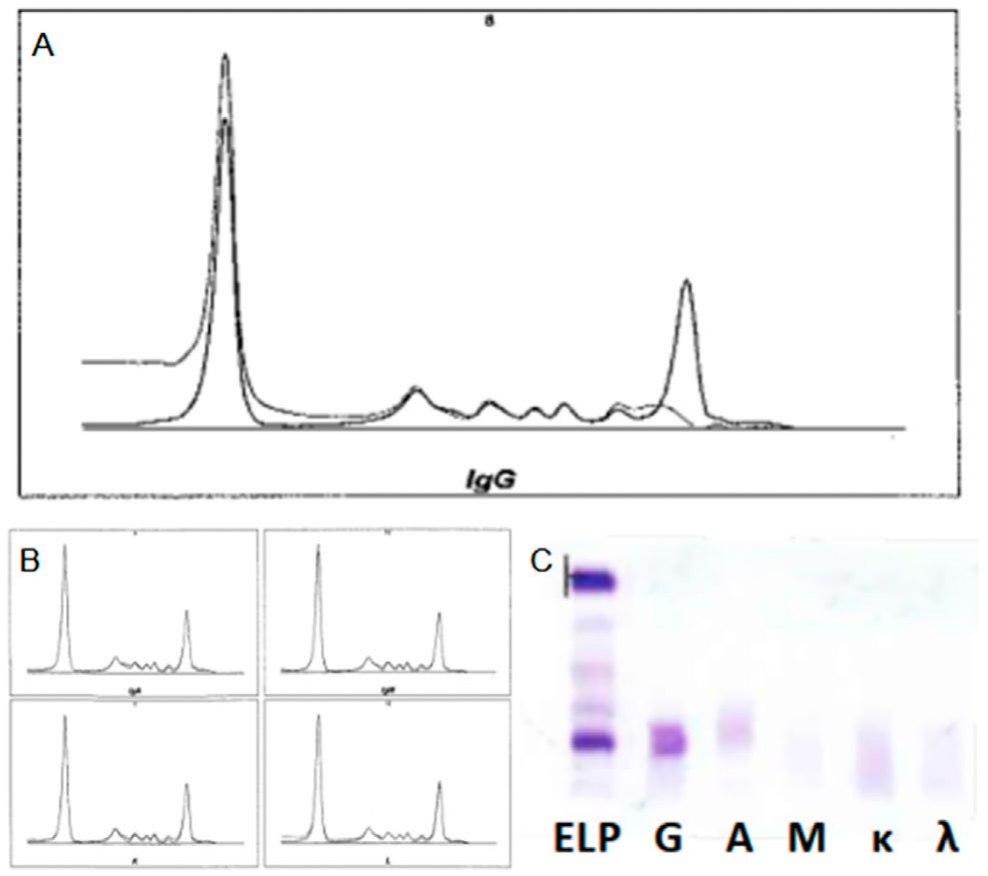
(A) Serum immunotyping displaying subtraction in the IgG window (B) with no subtraction from other antisera. (C) Serum immunofixation displaying a distinct monoclonal band in the anti‐γ lane, with no band in the anti‐κ or anti‐λ lanes. In lane G, a halo of immunofixed protein surrounds the artefactual clearing in the center of the band, a typical case of antigen excess.

Then, immunotyping (IT) was performed by the same instrument. As shown in (Figure 1A), subtraction appeared only after the addition of IgG antiserum, with no equivalence in any of the light chains' windows (Figure 1B). Thus, all other patterns were comparable to the reference trace.

Such an outcome is sufficient to determine γ‐immunoglobulin isotype, yet further examination was pursued to better investigate the apparent lack of LCs. Accordingly, serum immunofixation, urine immunofixation, and quantitative tests aimed at immunoglobulins were performed.

Both serum and urine IFE were executed on the Sebia HYDRASys 2 instrument with the adoption of Sebia HYDRAGEL 9IF and 9BJ kits, respectively. Figure 1C displays the appearance of a distinct monoclonal band in the anti‐γ lane of the serum IFE plate, with no correspondence in either anti‐κ or anti‐λ lanes.

**FIGURE 2:**
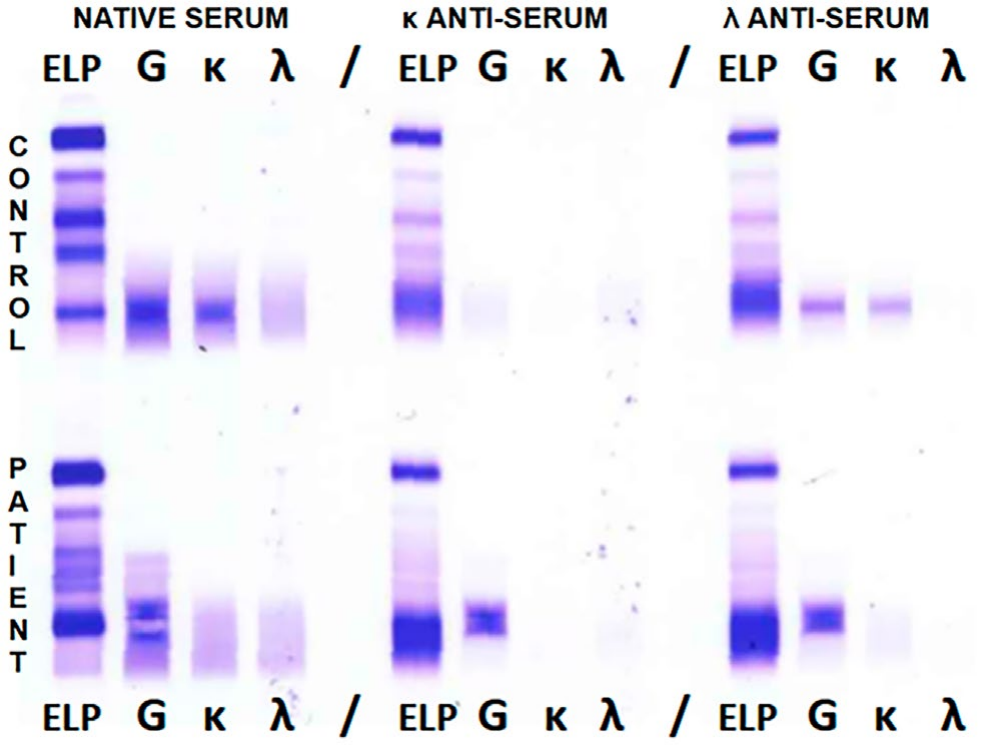
Urine IFE displaying a distinct monoclonal band in the anti‐GAM lane, with no band in either bound or free anti‐κ and anti‐λ lanes. A halo of immunofixed protein surrounds the artefactual clearing in the center of the band, a typical case of antigen excess.

The urine IFE plate presents lanes with antisera aimed at bound LCs and FLCs. Yet, it showcased only the serum's same mobility monoclonal band in the anti‐GAM lane, with no aspects of glomerular damage, considering the lack of albumin or other glomerular proteins in the ELP lane (Figure 2). Finally, the patient's urine was subjected to a serum IFE protocol to evaluate the findings from the anti‐GAM lane separated into the three main isotypes, confirming the γ‐isotype of the urine's monoclonal band.

Next, further investigations were performed to quantify immunoglobulin γ‐α‐μ isotypes, free light chains (FLCs), and IgG subclasses. The main immunoglobulin isotypes were first analyzed with Siemens Atellica CH Analyzer immunoturbidimetry (Siemens AG, Munich, Germany), while IgG subclasses, FLCs‐κ, and FLCs‐λ were analyzed with Siemens BNII nephelometry.

Table 2 shows the quantitative results from the same sample, analyzed on days 3 and 4 of hospitalization.

**TABLE 2:** Quantitative tests results.

	Atellica	BNII	RR
Serum			
SCr	0.9 mg/dL		0.7–1.2 mg/dL
TP	4.4 g/dL		5.7–8.2 g/dL
Alb	2.7 g/dL		3.2–4.8 g/dL
IgM	0.7 g/L		0.5–3.0 g/L
IgA	1.2 g/L		0.4–3.5 g/L
IgG	27 g/L	24.6 g/L	6.5–16.0 g/L
IgGSum		48.4	
IgG1		0.79 g/L	3.6–10.27 g/L
IgG2		0.41 g/L	0.81–4.72 g/L
IgG3		47.2 g/L	0.14–1.06 g/L
IgG4		0.01 g/L	0.05–1.98 g/L
FLCs‐κ		21.5 mg/L	3.3–19.4 mg/L
FLCs‐λ		35.4 mg/L	5.7–26.3 mg/L
κ/λ ratio		0.61	0.26‐1.65
Urine			
TP	0.9 g/24 h		< 0.15 g/24 h
LCs‐κ		4.85 mg/dL	< 0.7 mg/dL
LCs‐λ		2.77 mg/dL	< 0.38 mg/dL

*Note:* Quantitative measurements from Siemens Atellica CH Analyzer immunoturbidimetry and Siemens BNII nephelometry.

Abbreviations: Alb, albumin; IgGSum, calculated sum of IgG subclasses; SCr, serum creatinine; TP, total protein.

The first element of interest was the paradoxical differential between IgGSum (48.4 g/L) and total IgG (24.6 g/L). Despite the reliability of both methods, IgG quantitation was later repeated on Siemens BNII to ensure comparability between measurements, confirming the previous result.

Such findings are likely connected to variations in the reagents' target epitopes and are possibly explained by an altered immunoglobulin structure [6]. Heavy chain isotypes' specific determinants are found on the Fc portion of the constant region, while IgG subclass specificity lies in the hinge region, next to a region susceptible to truncation. Both regions are targeted by the detection antibodies of the abovementioned methods. Hence, the immunoglobulin truncation may portray a role, but it is not possible to exclude other motivations [6]. Such observations raise intriguing questions about the underlying immunoglobulin structure.

Assuming the reliability of the SPE assessment, the IgG value seems closer to the computed calculation via perpendicular drop. The correlation between the latter, measured at 12.9 g/L, and the IgG in nephelometry (24.6 g/L) shows a twofold difference. If, alternatively, the M‐spike was represented by the IgG3 value (47.2 g/L), this would reveal a 3.6‐fold difference, possibly being less plausible.

### 2.2 Immunoselection Test

γ‐HCD diagnosis can be missed because of low‐to‐moderate heavy chain concentration. In other cases, the definitive absence of LCs may be difficult to distinguish from poor reactivity to antisera. Thus, the basic protein laboratory tests are not sufficient for a definitive diagnosis of HCD, and direct methods such as immunoblot or immunoselection are needed to completely exclude the presence of light chains [6]. To appease any concern of poor detection or weak staining linked to lower avidity and affinity, we furthered our investigation by combining the patient's serum with specific antisera in an experimental immunoselection plate.

Initially, we proceeded by selecting a control serum from a patient with a similar monoclonal presentation to our case. All quantitative and qualitative properties were comparable, except for the control, which presented an M protein made of IgG‐κ.

Both sera were mixed in two aliquots, represented by a 1:10 ratio of serum to anti‐κ and anti‐λ antisera, respectively. The four mixtures were incubated for 48 h at 4°C, then underwent centrifugation for 10 min at 10 900 rpm. The supernatant was collected to perform immunofixation. Gel of choice was Sebia HYDRAGEL 2–4IF, exposed to antisera from a Bence Jones kit. These antisera were chosen for higher avidity than other kits, enhancing the finding of a plausible light chain. Yet, even if running a Bence Jones migration program could have been preferred for the same reason, the longer incubation with the antisera was too excessive for this case (*figure not shown*). Hence, we suggest a shorter program when facing a well‐represented HCs or M protein and a longer one for lower quantities.

**FIGURE 3:**
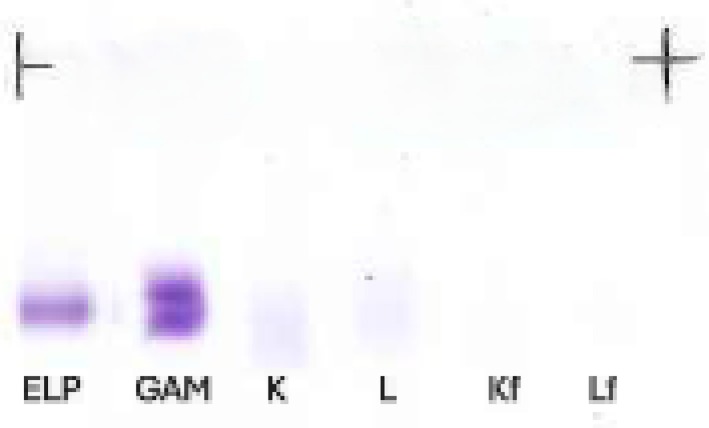
Control's (upper row) and patient's (lower row) sera immunoselection on Sebia HYDRAGEL 2–4IF, exposed to antisera from the Bence‐Jones kit and 2/4 IF MS/MD migration program from Hydrasys 2 (Sebia, Lisses, France). Three conditions are represented: Non‐diluted serum (left section, lane 2–5), anti‐κ antiserum incubated with supernatant (central section, lane 7–10), anti‐λ antiserum incubated with supernatant (right section, lane 12–15).

The plate was designed in 6 sections, as both patient's sera were investigated in three different conditions (Figure 3). First, non‐diluted serum, followed by the supernatant incubated with anti‐κ antiserum; finally, the supernatant incubated with anti‐λ. Each section was then represented by the same four lanes: ELP, anti‐γ, anti‐κ, and anti‐λ.

As shown in Figure 3, both antisera did not bind the HCs in the case with suspected γ‐HCD, since the band remained well‐represented and constant in all three conditions, always appearing in the ELP and γ lanes. Additionally, the polyclonal background shown in the lanes not bound by the opposite LCs antiserum proved the correct execution of the method. Conversely, in the patient with IgG‐κ monoclonal gammopathy, the component bound the anti‐κ antiserum and precipitated, not appearing in the central section. Instead, it emerged in the ELP, γ, and κ lanes of the other two conditions. Finally, a mixture of the suspected γ‐HCD serum in a 1:40 ratio with anti‐γ antiserum removed the band from the IF plate, further proving the IgG isotype (figure not shown).

What can be deduced is that in the case of a gammopathy with low affinity to light chains, as opposed to a real HCD, the antiserum would have precipitated with any component during incubation. Thus, any monoclonal band would have disappeared in one of the two LC mixture‐related conditions. As a result, in our patient, the immunoselection plate demonstrated the absence of a link between light and heavy chains, ruling out the hypothesis of a gammopathy with difficult LCs identification. Besides, the anti‐γ lane of the patient's immunoselection revealed two additional bands on top of the main monoclonal band, especially in the non‐diluted serum. This finding was later investigated by performing a molecular weight separation gel (HYDRAGEL 5 Proteinurie).

### 2.3 Molecular Weight Separation Gel

The first Proteinurie run (figure not shown) was executed according to the standard protocol. The plate disclosed a distinct band of 160 kDa. This finding generated further doubt since complete γ‐immunoglobulins weigh between 150 and 170 kDa, yet no LCs antiserum linked to the targeted HC. After ruling out N‐glycosylation and a longer hinge region of IgG3 as potential explanations for such a phenomenon, we speculate that the observed molecular weight might be due to a plausible unusual polymerization of the truncated HCs, also potentially hindering the quantitation of IgG3 and IgG. We emphasize that this remains a hypothesis, as a direct experimental confirmation could not be performed within our laboratory.

This led us to perform a second investigation using the Proteinurie gel, following serum incubation with antisera, as for the immunoselection. The preparation required a 1:20 dilution of the patient's serum to isotonic saline, followed by separation into three aliquots and a 1:10 ratio of mix to anti‐γ, anti‐κ, and anti‐λ antisera. After the same incubation and centrifugation of the immunoselection protocol, the supernatant was collected to perform a standard Proteinurie program. Unexpectedly, some elements produced a homogeneous background that hindered the interpretation of the results. Such interference diminished but did not disappear after removal of the kit Diluent required by standard protocol. Despite the curiosity to further investigate this case, we faced the method's limitations, which were not yet explored for a use other than that intended.

Finally, despite the targeted treatment, sepsis developed in an immunocompromised host who passed away 14 days after admission.

## 3 Discussion

γ‐HCD is a rare plasma cell neoplasm first described in 1964 by Franklin et al. [2], typically affecting individuals in the sixth to seventh decade of life, with a slight female predominance [7]. The diagnosis relies on detecting a clonal truncated γ heavy chain with no bound light chain [4].

γ‐HCD overall presentation is extremely diverse, with no set standard signs or symptoms [1]. In this case, protein analyses and lymph node biopsy provided definitive confirmation, while renal biopsy and bone marrow examination were not required due to preserved renal function (serum creatinine 0.9 mg/dL) and the absence of osteolytic lesions. Consequently, the literature reports multiple diagnostic pathways, reflecting the rarity of the disease and the low probability of having diagnostic criteria soon. Currently, there is no preferred approach in either diagnosis or treatment. The aggressiveness of the disease and the underlying neoplasm must be considered while developing a treatment strategy. Moreover, since the clinical outcome in most cases is hardly predictable, the prognosis may vary [1].

Our patient's overall presentation appeared complex due to the simultaneous presence of γ‐HCD, AITL, and reactivation of EBV infection. Although the real association between γ‐HCD and AITL is still under review, [8] EBV lymphomagenic effect is involved in the development of a great variety of lymphoproliferative conditions, including AITL, [9] and its association with γ‐HCD is also described [10]. It is under debate whether AITL's distinctive immune system impairment might play a role, and how the complex interplay with EBV's activity could affect γ‐HCD and AITL etiopathogenesis. Besides, it is well acknowledged that immunoglobulin's heavy chain expression is often altered by EBV's activity, especially with immune deficiency or immune dysregulation [11]. Although a direct pathogenic link between EBV infection and γ‐HCD cannot be established, EBV is known to promote clonal B‐cell expansion, interfere with immunoglobulin gene rearrangement and expression, and contribute to a broad spectrum of lymphoproliferative disorders [11]. These mechanisms may provide a biologically plausible explanation for the coexistence of EBV infection, AITL, and γ‐HCD in the observed case, despite the absence of prior reports demonstrating a causal association. Yet, one more topic of discussion is how to rule out EBV as a possible source of interference in LC's expression.

The diagnostic issue is also controversial: in our case, HCD was revealed by capillary zone electrophoresis associated with IT and later confirmed by an experimental immunoselection plate. Although past literature [4, 6, 12, 13] shows a broad list of methods used to characterize an unbound HC directly, we consider our approach to be reliable. Still, the debate on how to directly address γ‐HCD's diagnostic features with the typical laboratory instruments has its validity. Indeed, the truncated HC and the apparent lack of LCs still constitute a diagnostic challenge.

Even with a low bone marrow disease burden, γ‐HCs are increasingly produced by clonal plasma cells or other lymphoid elements [14]. The molecular weight of γ‐HCs, usually between 27 and 49 kDa, may vary due to truncation and configuration in monomeric or dimeric forms [10].

Despite completely lacking the CH1 domain, truncated γ‐HCs are not retained intracellularly as expected. The lack of the CH1 domain, in charge of LCs binding, stops HCs proteasome degradation via the HSP‐78 pathway [4]. Some authors suggest CH1 truncation in Heavy Chain Deposition Disease might consist of a pro‐survival event implemented by plasma cells, since the intracellular retention of a full‐length HC would be toxic to the clone [14]. In addition, the cells expressing γ‐HCs might acquire a growth advantage, as the altered chain could enable antigen‐independent aggregation and BCR signalling [4].

Part of the diagnostic challenge lies within the truncation itself: the lower molecular weight of the HCs causes renal clearance, decreasing serum concentration. Consequently, it could be challenging to determine the absolute absence of LCs. Additionally, some monoclonal components might exhibit low affinity to specific antisera, making it difficult to discriminate between the two conditions [4]. Furthermore, due to low serum concentration, CZE shows no monoclonal spike in 20%–40% of profiles; hence, the diagnosis is easily overlooked [7].

To overcome these obstacles, the most common solution is combining serum and urine IFE with SPE, IT, and quantitative tests. Although such techniques cannot demonstrate either the structural abnormality of the HCs nor the absence of bound LCs, direct methods are not always available, and many laboratories may face technical and organizational difficulties. In such instances, immunoselection proves useful, as it requires standard tools, enabling in‐house application by any IFE‐skilled laboratory.

Among the indirect findings, one of the most reliable is the combination of an isolated heavy chain band in serum and urine IFE with neither elevation in quantitative LCs measurements. Yet, the clinical pathologist must be aware of how this combination does not rule out a monoclonal gammopathy with low‐reacting LCs.

After this case, to ensure the reliability of the new method for future cases that may require a more in‐depth analysis, we designed the experimental immunoselection plate.

In conclusion, this work aims to highlight the diagnostic challenges of γ‐HCD, emphasizing the importance of a comprehensive, multidisciplinary laboratory approach along with advanced techniques for accurate identification and management. For laboratories facing difficulties in integrating direct methods, the best solution is a combination of the main investigations of the protein analysis section, possibly followed by immunoselection. A rare condition like γ‐HCD, especially presenting complex comorbidities, requires a meticulous and integrated diagnostic strategy to ensure an accurate differential diagnosis together with timely and effective patient care.

## Conflicts of Interest

The authors declare no conflicts of interest.

## Data Availability

The data that support the findings of this study are available on request from the corresponding author. The data are not publicly available due to privacy or ethical restrictions.
